# Weighing up Open Access Publishing in Nephrology—Bronze, Platinum, or Fools' Gold?

**DOI:** 10.34067/KID.0000000000000282

**Published:** 2023-10-19

**Authors:** Madelena Stauss, Lauren Floyd, Alexander Woywodt

**Affiliations:** Department of Renal Medicine, Lancashire Teaching Hospitals NHS Foundation Trust, Preston, United Kingdom

**Keywords:** open access, publication fees, article processing charges, publishing

## Introduction

Open access (OA) publishing describes a set of principles to provide access to research outputs free of charge or barriers.^1^ The traditional model of scientific publishing funded through subscription is now evolving into a landscape where authors and institutions pay publishers substantial article processing charges (APCs). Somewhat surprisingly, there has been very little attention to this topic in nephrology nor any debate of the implications. This piece aims to stimulate discussion from a variety of perspectives on the basis of both evidence and experience in the research field.

## The Evolution of OA Publishing

Historically, most high-impact research has been behind pay walls accessible through subscription. The OA movement emerged in the early 1990s, with key events such as the Budapest OA Initiative and the Berlin Declaration shaping its development.^1^ OA has exponentially grown since then, driven partly by the severe acute respiratory syndrome coronavirus 2 pandemic.^2^ This humanitarian aspect is one of the compelling reasons for OA, ensuring wider access and knowledge dissemination, benefiting the larger society. There are suggestions that all funded research should be OA only by 2025,^3^ and recent actions, such as the White House Office of Science and Technology Policy, require federally funded research to be freely accessible at publication without delay.^4^ Online OA negates the need for lengthier paper-based publication and dissemination, exemplifying one of the benefits OA provides.

At its inception, OA was proffered to be achievable through self-archiving,^1^ which is now described as “Green” OA when it occurs through approved repositories or personal websites. Similar to this is preprint publishing, which allows researchers to freely share their work with the scientific community regardless of institutional affiliations or subscription access. “Bronze” OA describes publishing where articles are free to read on the publisher page but lack a clearly identifiable license. In comparison, “Gold” OA is the more common approach at present where the publisher makes an article freely available online, usually after charging APCs. “Diamond” OA denotes an approach where costs are covered by institutions, societies, or organizations^1^; however, globally, there is a lack of concordance between eligibility and conditions for funding, leading to confusion and inequitable opportunities. Larger organizations, such as the United Kingdom funding body the Wellcome Trust, often require the immediate release of funded research work and covers APCs; however, other smaller charities and research organizations do not or cannot afford to. This can lead to money being diverted from research itself to cover publication costs, raising ethical questions.

Despite cautions against expecting individuals to absorb their own costs,^1,3^ some journals now charge in excess of $10,000 for an original article,^5^ the equivalent of two and a half years of a PhD student's salary in countries such as Brazil.^6^ Rising APCs are becoming unaffordable even in developed countries, hindering publication opportunities for students, early career researchers, or those lacking formal funding. Escalating fees run the risk of reducing research contributors, marginalizing already underrepresented groups, introducing bias, and limiting critical debate.^7,8^

The rise of OA has fueled predatory journals, jeopardizing research integrity for financial gain. Furthermore, the emphasis placed by some institutional or grant body review panels to publish in high Impact Factor journals may also perpetuate the ability to charge high APCs. The use and role of Impact Factors is in itself debated, which some argue is also “predatory” and is a much larger discussion than this perspective allows. Distribution of funds and access to grants varies between institutions and countries, with smaller organizations struggling to cover the ever-growing costs associated with OA publishing. In the United Kingdom, the Research Excellence Framework assesses the quality of research in higher education institutions; however, only OA papers are considered. This increases pressure on smaller, less well-funded institutions to pay the associated fees before their research can be recognized by health education funding bodies.

**Figure 1:**
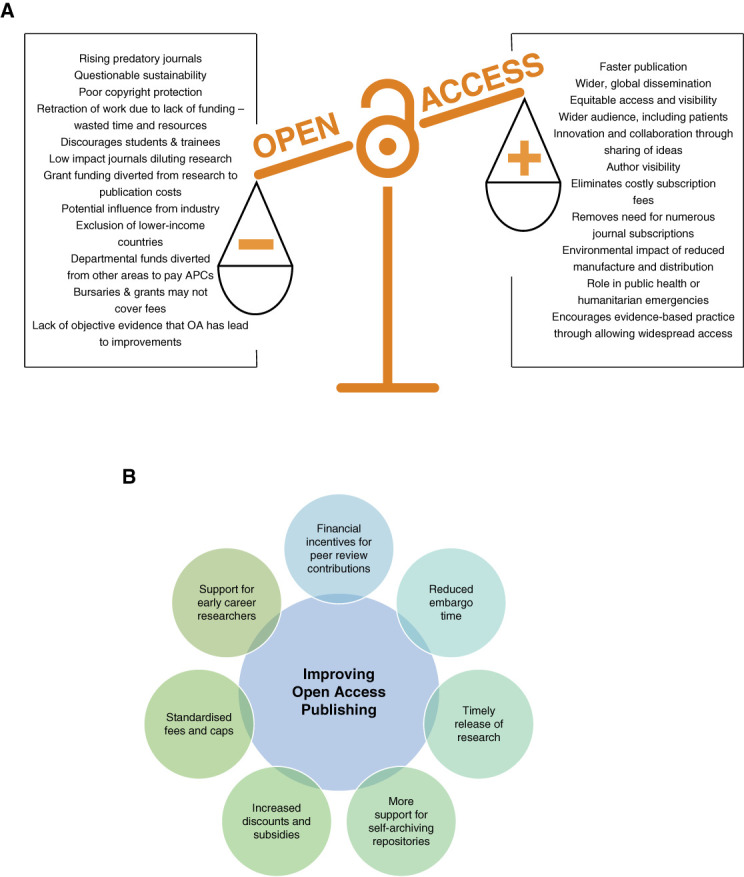
**Weighing up OA publishing and proposed solutions to overcome existing barriers**. (A) Current advantages and disadvantages of OA publishing and (B) proposed solutions. OA, open access.

Conversely, it is worth noting that while potentially expensive, OA journals eliminate subscription costs and the need for multiple subscriptions which can be beneficial to institutions and individuals. Figure 1A further presents advantages and disadvantages of OA publishing.

## The Current Landscape of OA in Nephrology

Currently, many nephrology journals adopt a hybrid approach whereby authors have the option of publishing either *via* the traditional subscription model (usually without fees) or OA. However, the majority are also “transformative,” belonging to publishing houses which have pledged to become fully OA in the near future as part of Plan S.^3^

Among the top 20 ranked nephrology journals according to the Scimago Journal Rank,^9^ 15 are hybrid, four OA only, and one subscription only, with most offering Gold OA with associated APCs. Some also provide the option of Green OA, which does not involve an APC, but articles have limited public domain availability until an embargo period has passed.

For Gold OA, current APCs range from $1800 to $4,300, excluding extra pages, color prints, and local taxes. However, journals often reduce or waive rates for authors from low- or middle-income countries (LMICs), associated societies, and on compassionate grounds. Many subscription-based journals offer free article availability to readers from LMICs or even patient groups, ensuring articles remain accessible with minimal or no reader cost.

The pressure on authors to cover rising APCs, and the publication costs for nephrology journals themselves, renders both parties vulnerable to industry or pharmaceutical influence. Despite nephrology being a physiologically focused specialty, the proportion of publications that are drug-related is steadily increasing, which has been suggested to be at the expense of basic and clinical science publications.^10^ Over recent decades, nephrology has had some of the fewest randomized controlled trials of all medical specialties, and funding by the National Institute for Health for kidney-related research is steadily reducing.^11^ While multifactorial, the perceived cost barrier, especially for those from independent centers or early in their careers, should not be underestimated as a deterrent to pursuing research.^12^ On a wider scale, the attrition in newly qualified doctors entering nephrology is widely acknowledged, with both early exposure and fostering an academic component found to be positive motivators for choosing nephrology.^13,14^ Portraying nephrology as an attainable, successful academic career option should be instigated early in students' experience with the removal of as many perceived deterrents as possible. Reducing the levy of APCs and an equitable publishing system that promotes high-quality, globally accessible and representative research would benefit authors and the nephrology community as a whole.

## Potential Solutions

The advantages of OA are clear, yet with ever-escalating APCs and Gold OA publication dominating the landscape, it is becoming unattainable or unsustainable for many. Urgent solutions need to be sought and are presented in Figure 1B.

Green OA provides the benefits of OA without high APCs. However, with most journals having an embargo period of 12 months by the time research is widely available, it is no longer contemporaneous. Shortening the embargo period would allow for the timely release of up-to-date research. More support should be available for self-archiving repositories, with championing of open archives. However, quality assurance is still needed, and fast self-archiving should not replace rigor or relevance.

Regarding journals, more transparency is required regarding what APCs contribute toward and the margin, or not, of any profits made. The Research Excellence Framework aims to provide accountability for public investments, and the same standards should be expected for journal investment. Currently, there is no regulation or cap on APCs. Therefore, those journals who wish to have low, or no, OA charges are disincentivized and their survival as a prospering journal may be threatened. Regulation to limit APCs would prevent huge disparities in practice and help to avoid the monetization of science.

APC subsidies exist, primarily benefiting authors from LMICs through discounts or waivers. We applaud this and urge journals to extend this to wider groups, such as students or authors from centers without dedicated funding, regardless of geographical location. Current systems favor established researchers affiliated with institutional support, often neglecting those at the start of their career. Existing organizations and societies could provide forms of sponsorship, which may encourage those to embark on research who would otherwise have been dissuaded.

Finally, the current peer review process relies on reviewers voluntarily providing their expertise and services. A credit system, where acknowledgment of peer review services contributes toward discounts in APCs, may not only help with the cost of publishing but also incentivizes those who would otherwise decline opportunities to peer review.

The advantages of OA are beyond reasonable doubt, but the resulting fees have significant implications for many. These unintended consequences are most certainly not what the initiators of OA publishing had in mind, and it comes as no surprise that The Budapest OA Initiative recently called for APCs to be abolished.^1^ For us, and many others, OA in its current form is a misnomer.^15^ OA resembles fools' gold, offering promises it currently cannot keep. Action is needed to uphold inclusivity and diversity as key principles of OA, preventing a future where solely funding but not novelty, hard work, or scientific rigor determine the fate of research in our specialty.
